# Selection and characterisation of Affimers specific for CEA recognition

**DOI:** 10.1038/s41598-020-80354-6

**Published:** 2021-01-12

**Authors:** Shazana Hilda Shamsuddin, David G. Jayne, Darren C. Tomlinson, Michael J. McPherson, Paul A. Millner

**Affiliations:** 1 The Leeds Bionanotechnology Group, School of Biomedical Sciences, University of Leeds, Leeds, LS2 9JT UK; 2 Department of Pathology, School of Medical Sciences, Health Campus, Universiti Sains Malaysia, 16150 Kubang Kerian, Kelantan Malaysia; 3 Bioscreening Technology Group, School of Molecular and Cellular Biology, University of Leeds, Leeds, LS2 9JT UK; 4 Astbury Centre for Structural and Molecular Biology, University of Leeds, Leeds, LS2 9JT UK; 5 Leeds Institute of Biomedical and Clinical Sciences, University of Leeds, Leeds, LS9 7TF UK

**Keywords:** Biological techniques, Biotechnology, Cancer, Cell biology, Molecular biology, Biomarkers, Molecular medicine

## Abstract

Carcinoembryonic antigen (CEA) is the only blood based protein biomarker at present, used for preoperative screening of advanced colorectal cancer (CRC) patients to determine the appropriate curative treatments and post-surveillance screening for tumour recurrence. Current diagnostics for CRC detection have several limitations and development of a highly sensitive, specific and rapid diagnostic device is required. The majority of such devices developed to date are antibody-based and suffer from shortcomings including multimeric binding, cost and difficulties in mass production. To circumvent antibody-derived limitations, the present study focused on the development of Affimer proteins as a novel alternative binding reagent for CEA detection. Here, we describe the selection, from a phage display library, of Affimers specific to CEA protein. Characterization of three anti-CEA Affimers reveal that these bind specifically and selectively to protein epitopes of CEA from cell culture lysate and on fixed cells. Kinetic binding analysis by SPR show that the Affimers bind to CEA with high affinity and within the nM range. Therefore, they have substantial potential for used as novel affinity reagents in diagnostic imaging, targeted CRC therapy, affinity purification and biosensor applications.

## Introduction

Colorectal cancer (CRC) is the fourth leading cause of cancer-related death and the third most commonly diagnosed malignancy worldwide^1^. Despite improvements in cancer treatment, advanced technologies in cancer diagnostics and augmentation of cancer awareness, the incidence and mortality rates of CRC still remain high. Late CRC diagnosis is associated with high morbidity and mortality rates. Hence, new strategies that can accelerate current diagnostic modalities in CRC diagnosis are crucial.

Carcinoembryonic antigen (CEA) is the first tumour associated antigen that was initially discovered in 1965 to be specific for colorectal cancer (CRC) and was later found to be abundant in many malignant tumours^2^. CEA, also known as carcinoembryonic antigen-related cell adhesion molecule 5 (CEACAM5) or CD66e, is the only blood based protein biomarker at present that is clinically approved as a prognostic biomarker for determining the treatment regime and as a surveillance biomarker for monitoring tumour recurrence in post-operative CRC patients^3,4^. CEA is predominantly overexpressed on the cell surface of tumours derived from epithelial origin including colorectal, lung, gastric, pancreatic and breast cancers and is also secreted into the blood stream^5,6^. High concentrations of CEA in the blood serum are commonly found in the advanced stages of CRC (stage II and III) and increased expression after surgery is an indicator of tumour recurrence or metastasis to the liver or lung^4,6^. Circulating CEA in the blood plasma is the prime target in enzyme-linked immunosorbent assay (ELISA) tests to determine the level of CEA and in assessment of cancer progression. CEA is a highly N-linked glycosylated oncofoetal antigen comprised of an N-terminal domain, three highly conserved repeat domains and a glycophosphatidylinositol (GPI)-membrane anchored hydrophobic C-terminal domain^5^.

For several decades, antibodies have been used as excellent natural binding reagents in therapeutic and diagnostic applications. They are known for their high specificity and sensitivity, strong affinity towards their target and long serum half-life^7,8^. The latter is a crucial feature of targeted drug carriers and biotherapeutic agents. However, their complex protein structure, batch-to-batch variability, expensive and slow production, complex chemical modification for oriented immobilisation in immunoassay applications and poor tissue penetration due to their size (~ 150 KDa)^9^ have rendered them of limited use in some biomedical and bioanalytical applications. These drawbacks have driven the generation of non-immunoglobulin protein scaffolds as alternative binding reagents such as Affibodies^10^, FN3 domains^11^, DARPins^12^, Anticalins^13^ and Affimers^14^.

Affimer proteins show comparable applicability to antibodies. They are engineered protein scaffolds (12–14 KDa) originally derived from a cystatin consensus sequence^15^ or human stefin A^16^. The former is commercially known as Affimer type II, and was used in this study, whilst, the latter is known as Affimer type I. These monoclonal reagents are small, monomeric, lack disulphide bonds and glycosylation sites and are thermostable^14,15^. The use of Affimers as versatile affinity reagents has been reported in broad applications targeting more than 350 different biological targets^14^ including in the modulation of protein function and protein–protein interaction^17–20^, directing formation of magnetic nanoparticles^21^ and development of highly sensitive and specific biosensors^22–25^. Although there have been several reports on the development of anti-CEA reagents from recombinant antibody technology^26,27^, based on the promising potential of Affimers as alternative affinity reagents, we postulated that generation of CEA binding Affimers could facilitate the acceleration of diagnostics and treatment of CRC and other cancers.

In this study, we describe the selection of Affimers specific to CEA protein screened from an Affimer phage display library. Each displayed Affimer comprises a cystatin-derived scaffold with two variable regions, each containing nine random amino acids, excluding cysteine, in each variable region. Through characterisation studies, we have demonstrated that the anti-CEA Affimers bind specifically and selectively to protein epitopes of CEA within complex protein matrices, such as cell lysates and in fixed cells. They recognise multiple forms of functional CEA including the fully glycosylated native form as well as incompletely glycosylated and deglycosylated forms. Kinetic binding analysis by SPR has shown that the Affimers bind to CEA with high affinities within the nM range. Therefore, they have potential as alternative bioreceptors in bioanalytical applications for CEA, notably in colorectal cancer.

## Results and discussion

### Phage display selection of Affimer proteins specific to CEA

**Figure 1:**
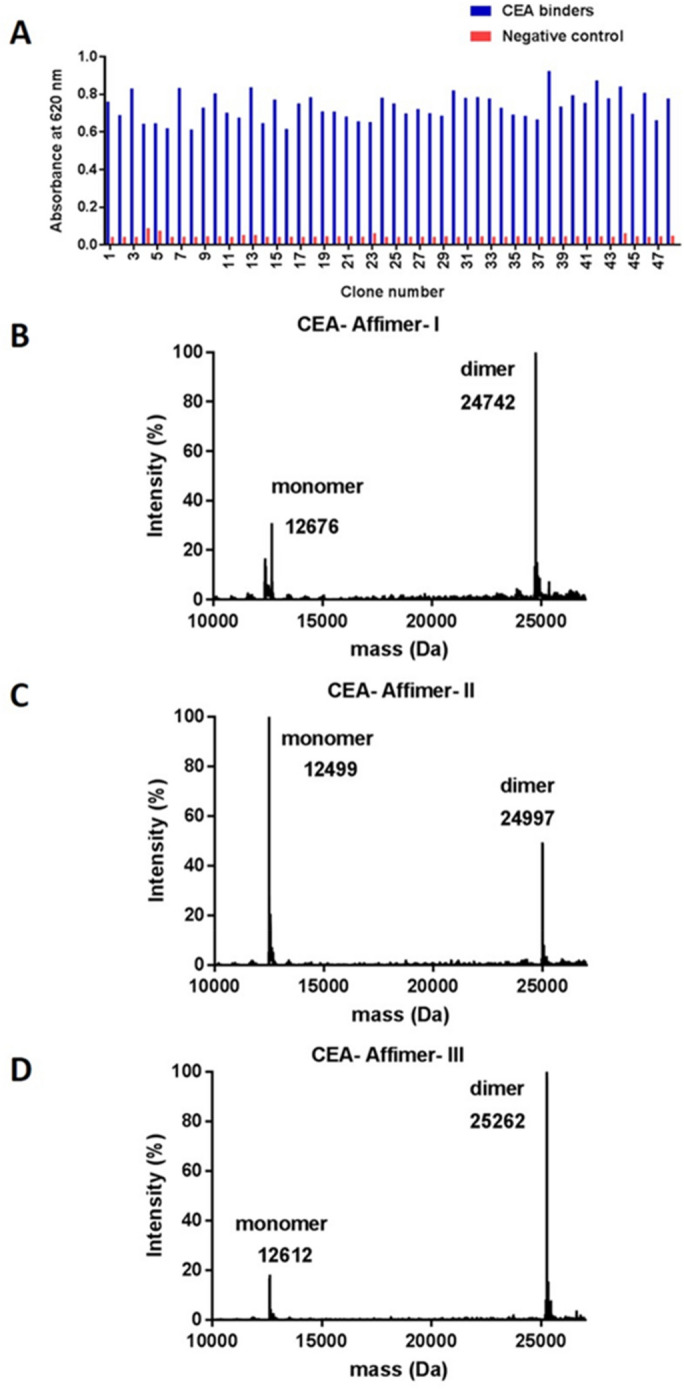
Phage ELISA of Affimer clones and LC–MS analysis of the purified CEA-Affimer protein. (**A**) Forty eight positive clones were incubated in wells coated with biotinylated CEA (blue bars) whilst biotinylated targets were omitted in the negative control wells (red bars); (**B**–**D**) the mass spectra of the purified CEA-Affimer I to III displaying the monomer and dimer peaks.

Affimers specific to CEA were identified from the phage display library constructed by the BioScreening Technology Group (BSTG), University of Leeds, UK. After three biopanning rounds, 48 randomly picked positive Affimer clones were evaluated for their binding ability to CEA by phage ELISA (Fig. 1A) and subsequently their DNA was sent for DNA sequencing to allow analysis of the binding loop sequences. Results revealed 3 unique CEA binders and amino acid analysis using ProtParam software suggested that the theoretical pI values of the Affimers were within the neutral range (pI ~ 7). CEA-Affimer-I and II contained 9 distinct amino acid residues in each variable region which are based on the design of the scaffold library reported by Tiede and co-workers^15^. However, CEA-Affimer-III has an extra amino acid residue in the variable region 2. CEA-Affimers II and III yielded 8.3 and 6.27 mg of protein from a 50 ml culture whilst CEA-Affimer-I only yielded 0.24 mg of purified protein. The purity and molecular mass of the Affimer proteins was determined using liquid chromatography mass spectrometry (LC–MS) analysis and results showed that all proteins were pure and the molecular masses of CEA-Affimer-I, II and III were 12,676, 12, 499 and 12,612 Da, respectively (Fig. 1B–D). Dimers were observed with CEA-Affimer-I and III which may have resulted from the formation of disulphide bond between thiol groups located at the C-terminus.

### Specificity of binding of Affimers on CEA-expressing cells

**Figure 2:**
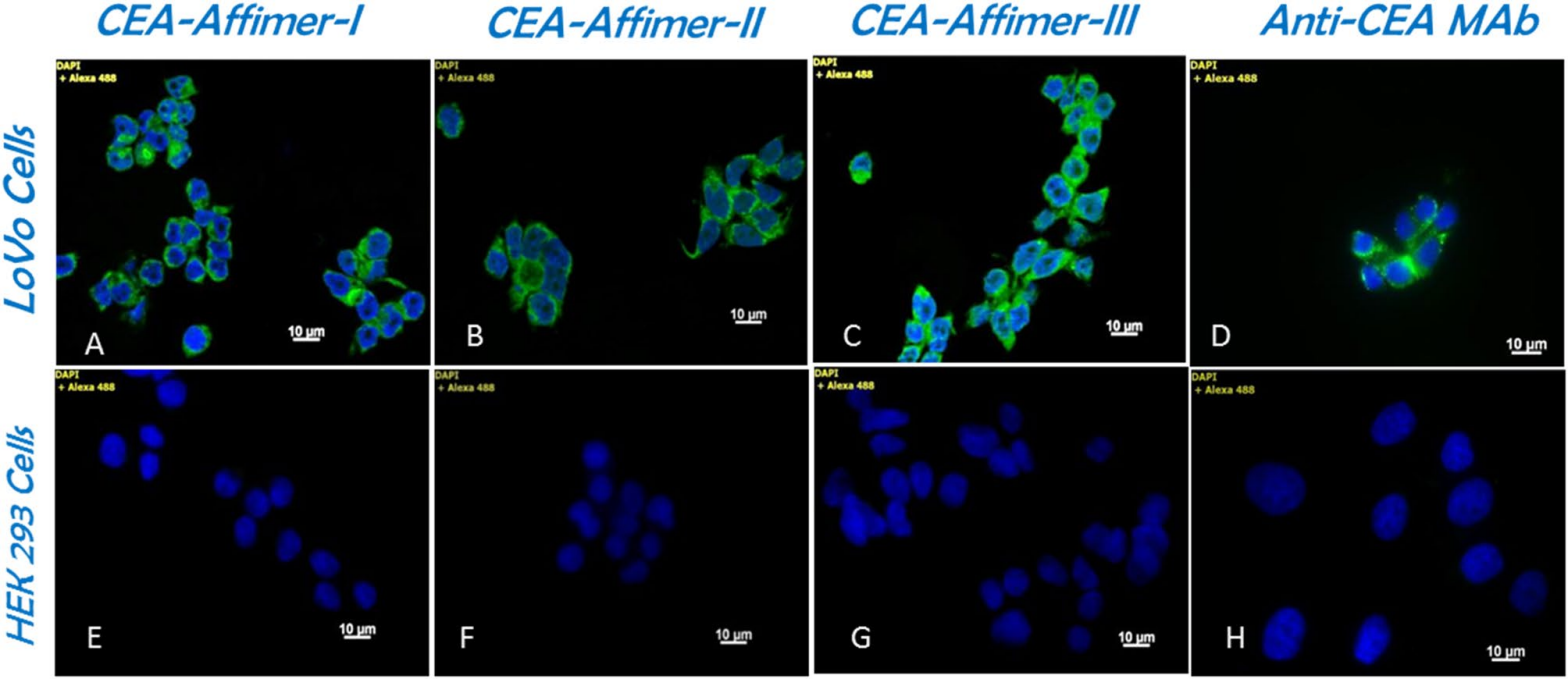
Cell binding and selectivity of anti-CEA Affimers on LoVo cells in comparison to monoclonal antibody. (**A**) to (**C**) show affinity-fluorescence staining of CEA binding Affimer I, II and III on LoVo cells compared to immunofluorescence staining using anti-CEA monoclonal antibody (**D**). No binding is present on corresponding HEK 293 negative control cells (**E** to **H**).

Phenotypically, LoVo cells are known to over-express CEA on their plasma membranes^28,29^. To determine the specificity of Affimer binding to CEA on fixed LoVo cells, direct fluorescent staining was performed by using biotinylated anti-CEA Affimers as the primary reagent and streptavidin conjugated DyLight 488 as the detection reagent. Immunofluorescence staining using monoclonal anti-CEA and Alexa Fluor 488 conjugated secondary antibodies was included as comparison. Localization of CEA was observed on the surface of LoVo cells which is consistent with CEA being a membrane–anchored glycoprotein (Fig. 2A–C) but no staining was found on HEK 293 cells (Fig. 2E–H). Interestingly, the Affimers generally showed higher intensity of staining with a wider distribution compared to immunostaining (Fig. 2D). These findings indicated that CEA binding Affimers have better sensitivity and specificity than the corresponding antibody. This could be due to their being physically smaller than antibodies (~ 3 nm for Affimer vs. ~ 14 nm for IgG) facilitating better penetration into fixed cells^14,30^.

### Specificity of Affimer binding to soluble CEA

**Figure 3:**
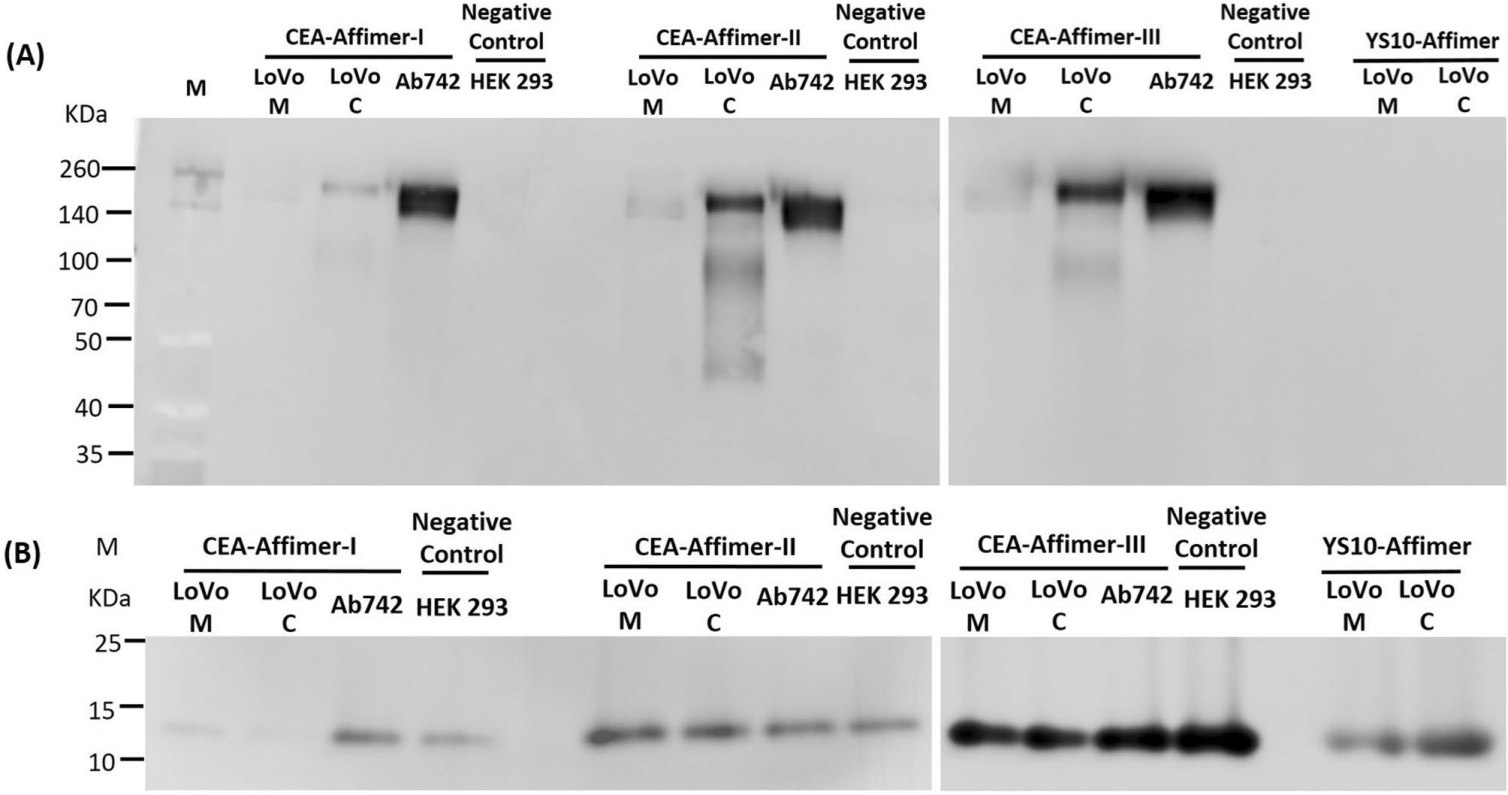
Immunoblotting of Affimer precipitated CEA. Monoclonal anti-CEA and anti-His_6_ tag antibodies were used to probe (**A**) the CEA protein and (**B**) Affimers, respectively from the pull-down complex. Yeast SUMO-10 binder was used as a control Affimer. Commercial CEA from Abcam was used as positive control and cell lysates from HEK 293 as negative control. LoVo M and C are samples from medium and cell lysates, respectively. Full-length blots are presented in Supplementary Fig. S3.

Apart from being overexpressed on colorectal cancer cells cell membranes, CEA is also secreted in soluble form into the blood stream which is predominant form of CEA used clinically to determine the staging of colorectal cancer and to monitor tumour recurrence. To investigate the ability of Affimers to bind specifically to native CEA in a complex protein mixture, pull-down assays were performed against the cell lysates and soluble CEA present in the culture medium. Purified Affimers were immobilized onto Ni^2+^-NTA resin which was subsequently used to pull-down CEA from protein mixtures. Results from SDS-PAGE analysis demonstrated that all anti-CEA Affimers specifically pulled-down fully-glycosylated CEA from the cell lysates and soluble CEA secreted into the media as shown by a single band detected around 200 KDa (Supplementary Fig. S1). An identical band was seen in reference CEA, commercially obtained from Abcam. By contrast, no band was detected from the cell lysates of HEK 293 cells, which was used as a negative control, except a single band at 13 KDa that corresponded to Affimer protein. Similarly, only a single band at Affimer size was observed when using yeast SUMO-10 binder as the control Affimer ligand (Supplementary Fig. S1). Immunoblotting of the corresponding pull-down complex using anti-CEA and anti-His_6_ tag antibodies further corroborated that the bands detected at 200 KDa and 13 KDa do indeed represent the CEA and Affimer proteins respectively, as shown in Fig. 3 and Supplementary Fig. S2. Taken together, these findings demonstrated that CEA binding Affimers were able to specifically bind to native CEA protein within the complex protein mixture.

### Recognition of CEA protein epitopes by Affimers

**Figure 4:**
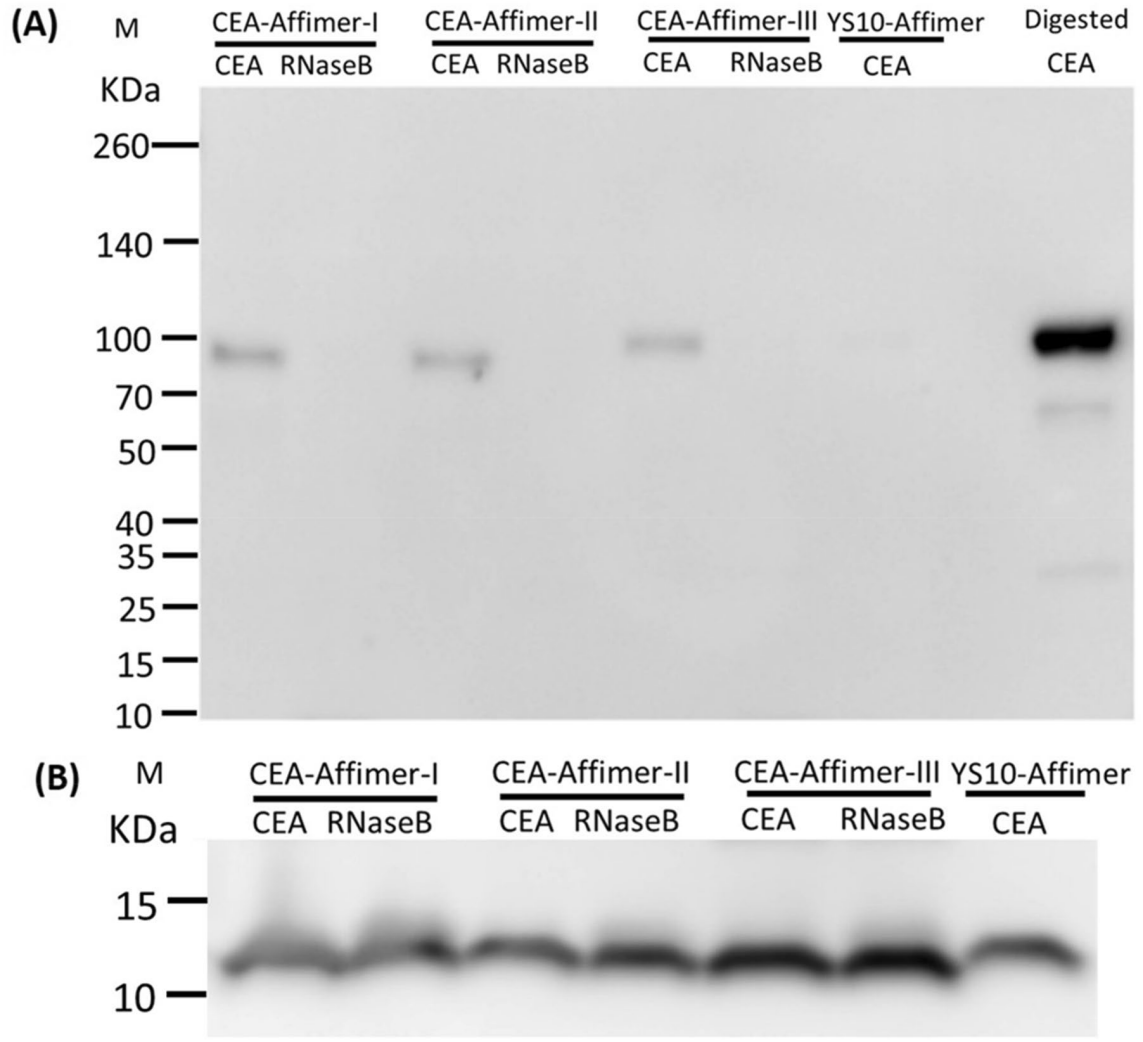
Immunoblotting of Affimer precipitated deglycosylated CEA. Immunoblotting of affinity-precipitation using CEA binding Affimers as ligand to pull-down deglycosylated CEA (n = 2). Monoclonal anti-CEA and anti-His_6_ tag antibodies were used to probe the pulled-down (**A**) deglycosylated CEA and (**B**) Affimers, respectively. Yeast SUMO-10 binder was used as a control Affimer and RNase B was used as non-specific analyte. Digested CEA was used as a reference to the pulled-down CEA in blotting. Full-length blot of Fig. 4B is presented in Supplementary Fig. S3.

CEA is a highly N-linked glycosylated protein comprising 60% oligosaccharide which is added during post-translational modification. Epitope mapping studies of CEA protein using monoclonal antibodies indicated that there are two types of epitope present on CEA; protein and sugar epitopes^31,32^. To distinguish whether CEA binding Affimers interacted with sugar or protein epitopes, deglycosylation of CEA protein via enzymatic PNGase F treatment was conducted. The same pull-down assay as previous was performed but this time challenged against deglycosylated CEA. RNase B protein was included as a positive control for PNGase F treatment and a non-specific target for CEA-Affimers. Yeast SUMO-10 binder was used as control Affimer. The pull-down complexes were separated by reducing SDS-PAGE and the extent of Affimer binding was investigated by immunoblotting. Results showed that the molecular weight of CEA was reduced to around 100 KDa after deglycosylation and all anti-CEA Affimers still bound specifically to this form (Fig. 4A). No binding was observed to deglycosylated RNase B and from the control Affimer. Additionally, an immunoblot probed using anti-His_6_ tag antibody confirmed the presence of Affimers in the pull-down complex as shown in Fig. 4B and Supplementary Fig. S3. Based on these findings, it was clear that CEA binding Affimers recognized protein epitopes of CEA and deglycosylation did not affect the binding interaction of anti-CEA Affimers. However, further work needs to be done to distinguish which protein epitopes do the anti-CEA Affimers bind to, as there are seven protein domains within CEA.

### Kinetic binding analysis using SPR

**Figure 5:**
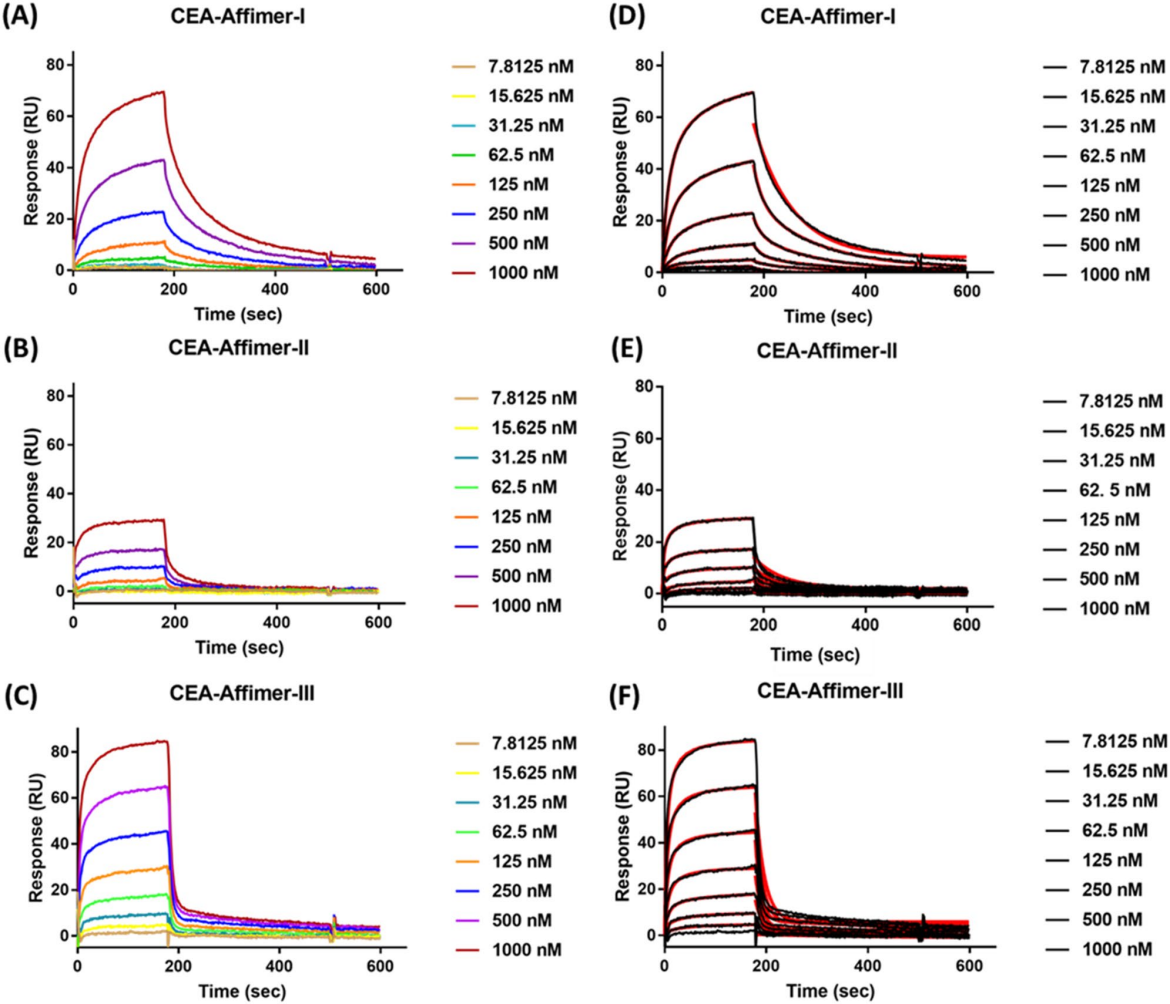
Kinetic binding analysis of CEA binding Affimers. Binding of CEA protein was tested at concentrations of 7.8 nM-1 μM using twofold dilutions on immobilized Affimer. (**A**) CEA-Affimer-I; (**B**) CEA-Affimer-II and (**C**) CEA-Affimer-III. Data were normalized using a double-referencing methods by subtracting the responses from an unmodified reference cell and blank injection using running buffer only. (**D**–**F**) Global fit analysis of the corresponding CEA/Affimer binding data using a simple 1:1 Langmuir binding interaction model. The experimental SPR trace (black lines) and theoretical fit (red lines) are overlaid and were used to extract the kinetic parameters of the interaction including the association (*k*_a_) and dissociation (*k*_d_) rate constants and equilibrium dissociation constant (*K*_D_).

To determine the kinetics of binding between Affimers and CEA, surface plasmon resonance (SPR) analysis was performed. A low-density surface was generated by immobilizing biotinylated anti-CEA Affimers as bioreceptors to give 270 RU (Supplementary Fig. S4) and the interaction with series of CEA concentrations (7.8125 nM to 1 µM) (Fig. 5A–C) was evaluated at high flow-rate (50 µl/min) to eliminate the mass transport limiting factor. The kinetic parameters were extracted from the global fitting of experimental data using a 1:1 Langmuir interaction model (Fig. 5D–F). Small χ^2^ value ranging from 0.066 to 1.94 indicated a very close-fit between the fitted curves (Fig. 5D–F, red lines) and the experimental curves (Fig. 5D–F, black lines). CEA-Affimer-I and II had similar association and dissociation rate constants. By comparison, CEA-Affimer-III had slower association and dissociation rate constants. Association (*k*_a_), dissociation (*k*_d_) and equilibrium (*K*_D_) constants are given in Table 1. CEA-Affimer-I had the highest affinity with *K*_D_ = 6.46 ± 1.38 nM, whilst CEA-Affimer-II and III showed moderate affinities with *K*_D_ = 15.3 ± 0.37 nM and 34.4 ± 16 nM, respectively.

**Table 1:** Kinetic values of CEA-Affimer interaction.

Receptor	*k*_a_ ± SE (M^−1^ s^−1^)	*k*_d_ ± SE (s^−1^)	*K*_D_ ± SE (nM)	χ^2^
CEA-Affimer-I	(2.00 ± 0.84) × 10^6^	(1.29 ± 0.13) × 10^–2^	6.46 ± 1.38	0.066
CEA-Affimer-II	(1.14 ± 0.84) × 10^6^	(1.75 ± 1.08) × 10^–2^	15.3 ± 0.37	0.049
CEA-Affimer-III	(6.78 ± 1.86) × 10^4^	(2.33 ± 0.26) × 10^–3^	34.4 ± 16	1.94

Previous studies have investigated the relationship between specificity and affinity of antibody and protein interactions. They concluded that the antibody paratopes are substantially enriched with amino acid residues containing aromatic side chain (tyrosine, tryptophan and phenylalanine) and surrounded by short-chain hydrophilic side chains (aspartate, asparagine, serine, threonine, and glycine) with the latter playing an important role in attaining the conformation that enables high affinity binding^33,34^. Interestingly, these amino acid residues (all except threonine) are present in each variable region of all anti CEA-Affimers with approximately 44% frequency (8 of 18 amino acids) within their variable regions. The anti- CEA-Affimers we selected exhibited high affinity constants within the nM range from 6.46 to 34.4 nM, also in agreement with this notion. Accordingly, Sha et al.^35^ postulated that this phenomenon occurs in the majority of synthetic binding proteins including Nanobodies and Monobodies whereby these residues provide favourable interaction to the epitope surface for the short period of phage display screening under strong selection pressure. In agreement with previous studies^36^, we also observed that higher affinity Affimers (Table 1) expressed less well and CEA-Affimer-I, with the highest affinity expressed at much lower levels than CEA-Affimer-II and III.

## Conclusions

This is the first report to demonstrate a novel affinity reagent generated against CEA-derived from a highly stable non-immunoglobulin Affimer protein scaffold. The successful results indicate the potential of Affimers to be exploited in diagnostic imaging as molecular probes for imaging CEA expressed by tumours. Introduction of a single cysteine residue in the C-terminal and His_8_-tag region, distant from the antigen binding site provides an extra advantage in theranostic applications potentially allowing tumour imaging and delivery of therapeutic agents in a single molecule since the Cys thiol group permits easy attachment. Increased expression of CEA in serum after tumour resection normally correlates with tumour recurrence and metastasis. The ability of Affimer to bind to soluble CEA suggests another use of Affimers as affinity reagents in anti-metastatic therapy. Finally, immobilization of biotinylated Affimer onto streptavidin coated chips in SPR analysis demonstrate the possibility of Affimers as biorecognition elements in the fabrication of biosensor devices for CEA detection such as electrochemical biosensors for the development of point-of-care devices. A key element for a highly sensitive and effective analyte detection with regard to biosensor fabrication is site-directed receptor immobilization. The ease of modification via the thiol group at the C-terminal or his_6_-tag region facilitate this with Affimer proteins. Finally, recent reports have cast doubts on the fidelity of antibody recognition in many reports^37,38^ here we show a fully validate affinity reagent, including the type of epitope recognised.

## Materials and methods

### Selection of Affimer proteins by phage display

CEA was sourced from Abcam and was biotinylated with a fivefold molar excess of EZ-Link NHS-SS-Biotin (Thermo Scientific) for 1 h at 20 °C and then desalted using Zeba Spin Desalting Columns (Thermo Scientific). The success of biotinylation was validated by ELISA (Supplementary Fig. S5). Three bio-panning rounds using one standard and two consecutive competitive-binding pans were carried out to screen for Affimer that bound as previously described^15^. Throughout the whole panning process, CEA and ySUMO were used as target molecule and positive control, respectively. Panning was performed on streptavidin coated wells for 1.5 h at 20 °C on a vibrating platform using the biotinylated CEA and ySUMO. Then, 10^12^ cfu pre-panned phage (phage preincubated in streptavidin-coated wells) were added into the biotinylated samples and incubated for another 2 h. Panning wells were then washed 27 times with 300 μl of PBST and eluted first with 0.2 M glycine, pH 2.2 for 10 min then neutralized with 1 M Tris–HCl, pH 9.1. A second elution followed with 0.1 M triethylamine for 6 min which was then neutralized with 1 M Tris–HCl, pH 7 prior to infection of ER2738 *E. coli* cells for 1 h at 37 °C. The infected cells were plated onto LB agar containing 100 μg/ml carbenicillin and grown overnight at 37 °C. Colonies were scraped and re-cultured in 2TY/100 μg/ml carbenicillin broth and subsequently infected with approximately 1 × 10^9^ M13K07 helper phage and grown overnight with 50 μg/ml of kanamycin at 25 °C and 170 rpm. The second and third pans were performed using competitive binding with streptavidin magnetic beads and with NeutrAvidin high binding capacity coated wells, respectively to increase the selectivity. The competitive procedure was the same as the first panning except that prior to elution of the bound phage, 2.5 µg of non-biotinylated target protein was mixed with 10 × blocking buffer, Halt Protease Inhibitor Cocktail and 80% (v/v) glycerol. The mixture was incubated overnight at RT washed, eluted and amplified as described above. To screen for positive clones, 48 randomly selected clones were tested for specific binding against the target protein via phage ELISA as previously described^15^. In brief, the phage ELISA was performed by incubating Affimers from the last panning round with or without biotinylated CEA. The associated DNA from positive clones were submitted for DNA sequencing analysis to identify the range of binders.

### Production of Affimer protein and purification

The coding region for selected Affimers was amplified by PCR using Forward primer (5′-ATGGCTAGCAACTCCCTGGAAATCGAAG) and Reverse primer (5′-TTACTAATGCGGCCGCACAAGCGTCACCAACCGGTTTG). During amplification, the use of a reverse primer containing an additional cysteine codon allowed subsequent generation of protein with a C-terminal cysteine. Double digestion with *Nhe*I-HF and *Not*I-HF restriction enzymes at 37 °C for 2 h of the PCR product and engineered *pET11a* vector were performed prior to ligation and the Affimer proteins were subsequently produced in BL21 (DE3) *E. coli* cells as previously described^15^. The cells were harvested, lysed and protein was purified using single step chromatography on Ni^2+^-NTA affinity resin.

### Biotinylation of Affimers

Purified Affimers were biotinylated at the C-terminal cysteine using biotin-maleimide. Affimer, 150 µl of 40 µM elution solution was mixed with an equal volume of washed Immobilized TCEP Disulphide Reducing Gel (Thermo Scientific) for 1 h at 20 °C on a rotator mixer. Freshly reduced Affimer was mixed with 27 µl of 2 mM biotin-maleimide and incubation continue for 2 h at 20 °C. Excess biotin was removed using Zeba Spin Desalting Columns.

### Pull-down assays

Twenty micrograms of purified Affimer were dialysed in 1 × PBS prior incubated with 40 µl of washed Ni^2+^ -NTA slurry for 90 min at 4 °C on a rotator. Affimer-loaded resins were washed once with 1 × PBS and mixed with purified CEA from Abcam, cell lysates from LoVo cells, CEA secreted into the medium or deglycosylated CEA after treatment with PNGase F. These mixtures were incubated overnight at 4 °C on a tube rotator. The resins were washed three times with 1 × PBS before resuspending in SDS sample buffer. SDS-PAGE and western blot analysis were conducted to determine the success of the pull-down assay.

### Affinity and immunofluorescence staining of CEA on LoVo cells

LoVo (ATCC-CCL229), a cell line that positively expresses CEA, was grown in F-12 Nutrient Mixture containing GlutaMAX-I supplemented with 10% (v/v) heat-inactivated foetal bovine serum, 50 U/ml of penicillin and 50 µg/ml of streptomycin at 37 °C under 5% CO_2_ humidity conditions. Culture medium was aspirated every 3 days and collected for CEA protein isolation, whilst the cells continued to grow as monolayer cultures until reaching confluency at 7 day in a 150 cm^2^ flask. Meanwhile, the HEK 293 (ATCC-CRL-1573), cell line that does not express CEA protein was grown in DMEM containing GlutaMAX-I supplemented with 10% (v/v) heat-inactivated foetal bovine serum.

To determine the specificity of Affimer binding to CEA protein, fluorescence staining of fixed cells was investigated. The cells were seeded at 3 × 10^5^/well on cover slips in 6-well plates until they reached about 70% confluency. The medium was discarded, followed by three times washing in PBS (pH7.4) and subsequent fixation in 4% (w/v) paraformaldehyde solution for 10 min at 20 °C. The fixed cells were washed again three times in PBS and blocked for 30 min at 20 °C in SuperBlock T20 (TBS) blocking buffer, pH7.4 (Thermo Scientific). Cells were incubated overnight at 4 °C with 10 µg/ml of biotinylated Affimer diluted in blocking buffer. Then, 1 µg/ml of mouse anti-human IgG CEA monoclonal antibody (MA5-14675, Thermo Scientific) was used as primary antibody and included as the positive control on separate coverslips. After overnight incubation, the coverslips were washed again three times with PBS and incubated with secondary detection reagents. Goat anti-mouse IgG conjugated with Alexa Fluor 488 (Invitrogen) and Streptavidin DyLight 488 conjugate were applied to cells probed with primary monoclonal antibody and biotinylated Affimer, respectively. The secondary detection reagent was diluted to 1 µg/ml in blocking buffer and incubated with the labelled cells for 1 h at 20 °C in the dark. Coverslips were repeatedly washed with PBS for three times and mounted with ProLong Gold Antifade reagent with DAPI present to label the nuclei. Primary antibody or biotinylated Affimer were omitted in the negative control samples. CEA protein localization was visualized using an Axio Observer Z1 microscope equipped with an Apotome system of structured illumination (Zeiss, Germany).

### Isolation of crude CEA protein

The collected medium was centrifuged at 3220×*g* for 5 min at 4 °C to remove cell debris. The supernatant was then concentrated using Amicon Ultra Centrifugal Filters (15 ml, 50 KDa cut-off, Millipore) for another 10 min at 5000×*g* and 4 °C prior to protein extraction. Once cells reached confluency, medium was discarded and cells were washed once with 1 × ice-cold Dulbecco’s PBS. Protein was extracted from LoVo cells and HEK 293 cells by lysis in 1 ml of mammalian cell PE LB lysis buffer (G-Biosciences) containing 10 µl of 100 × Mammalian ProteaseArrest inhibitor cocktail (G-Biosciences) and incubated for 10 min at 4 °C. Cell lysates were then centrifuged at 16,000×*g* for 30 min at 4 °C and stored at -80 °C until further analysis.

### Deglycosylation of CEA protein using PNGase F

Deglycosylation of CEA protein using PNGase F was performed under denaturing conditions. RNase B protein was used as a positive control for PNGase F treatment. Generally, 20 µg of purified CEA or RNase B was heat denatured at 100 °C for 10 min in 1 × glycoprotein denaturing buffer (0.5% SDS, 40 mM DTT) according to the manufacturer’s protocol (New England BioLabs Inc). Samples were subsequently treated with 500 U/µl of PNGase F in 1 × GlycoBuffer 2 and 1% (v/v) NP-40 and incubated for 1 h at 37 °C. The extent of deglycosylation was visualized via SDS-PAGE and the deglycosylated protein was subsequently used for pull-down assays.

### Immunoblotting

Identification of CEA present in the cell lysates, protein secreted into the media and deglycosylated samples were investigated by immunoblotting. Proteins were separated via SDS-PAGE and transferred onto 0.2 µm pore PVDF membranes by applying a constant voltage of 100 V for 90 min with cooling. Membranes were blocked for 30 min in SuperBlock T20 (TBS) blocking solution and washed three times with 0.1% (v/v) TBST and subsequently probed with appropriate detection agents. These were either anti-CEA monoclonal antibody or anti-His_6_ tag-HRP conjugated antibody diluted to 1:1000 in blocking solution and incubated for 1 h at RT. For the membrane probed with anti-CEA monoclonal antibody, incubation of secondary HRP-conjugated antibody (1:2000) was performed for 1 h at 20 °C prior to the final washes. Three final washes were performed in 0.1% (v/v) TBST followed by three washes in 1xTBS before adding ECL substrate and imaging using a Syngene G:BOX imager.

### SPR analysis of Affimer binding kinetics

Kinetic binding analysis was conducted using a BIACore 3000 instrument (GE Healthcare, Sweden). The instrument temperature was set at 25 °C throughout the experiments and was cleaned by priming with running buffer containing 1 × PBS supplemented with 0.1% (v/v) Triton-X-100. A new streptavidin (SA) sensor chip (GE Healthcare, Sweden) was preconditioned by injecting 1 M NaCl and 50 mM NaOH at a flow rate of 40 µl/min prior to immobilization of biotinylated Affimer. Three flow cells (flow cell 2 to 4) were used to immobilize a low-density surface (230–270 RU) at a flow rate of 5 µl/min using 10 nM of biotinylated Affimer-CEA-I, II and III, respectively. Flow cell 1 was kept unmodified and used as a reference cell. CEA analyte was prepared in concentrations from 1 µM to 7.81 nM via twofold serial dilutions. Running buffer was flowed over the flow cells to equilibrate them prior to a succession of injections of CEA at increasing concentrations using a flow rate of 50 µl/min for 3 min. The dissociation rate of the CEA/Affimer complex was then monitored for 5 min followed by surface regeneration via injecting 1 × PBS containing 1 M NaCl for 4 min before injecting the subsequent CEA concentration. Experiments were done in triplicate. Data were normalized using a double-referencing methods by subtracting the responses from an unmodified reference cell and blank injection using running buffer only. Kinetic binding analysis was carried out using BIAevaluation software version 3.2 using a simple 1:1 Langmuir binding interaction model to determine the association and dissociation rate constants (*k*_a_ and *k*_d,_ respectively) and equilibrium dissociation constant (*K*_D_). These analyses are based on a combination of numerical integration and non-linear least-squares global curve fitting as previously described^39^.

## Supplementary Information

Supplementary Figures.
