# Supplementary material for: Selection and characterisation of Affimers specific for CEA recognition
Source: Sci Rep. 2021 Jan 12;11:744. doi: 10.1038/s41598-020-80354-6 (PMC7804248; doi:10.1038/s41598-020-80354-6)
Supplement: Supplementary file 1 — Supplementary Figures. [file 41598_2020_80354_MOESM1_ESM.pdf]

Selection and characterisation of Affimers specific for CEA recognition

Shazana Hilda Shamsuddin[1,2*], David G. Jayne[5], Darren C. Tomlinson[3, 4], Michael J. McPherson[3, 4] and Paul A. Millner[1*]

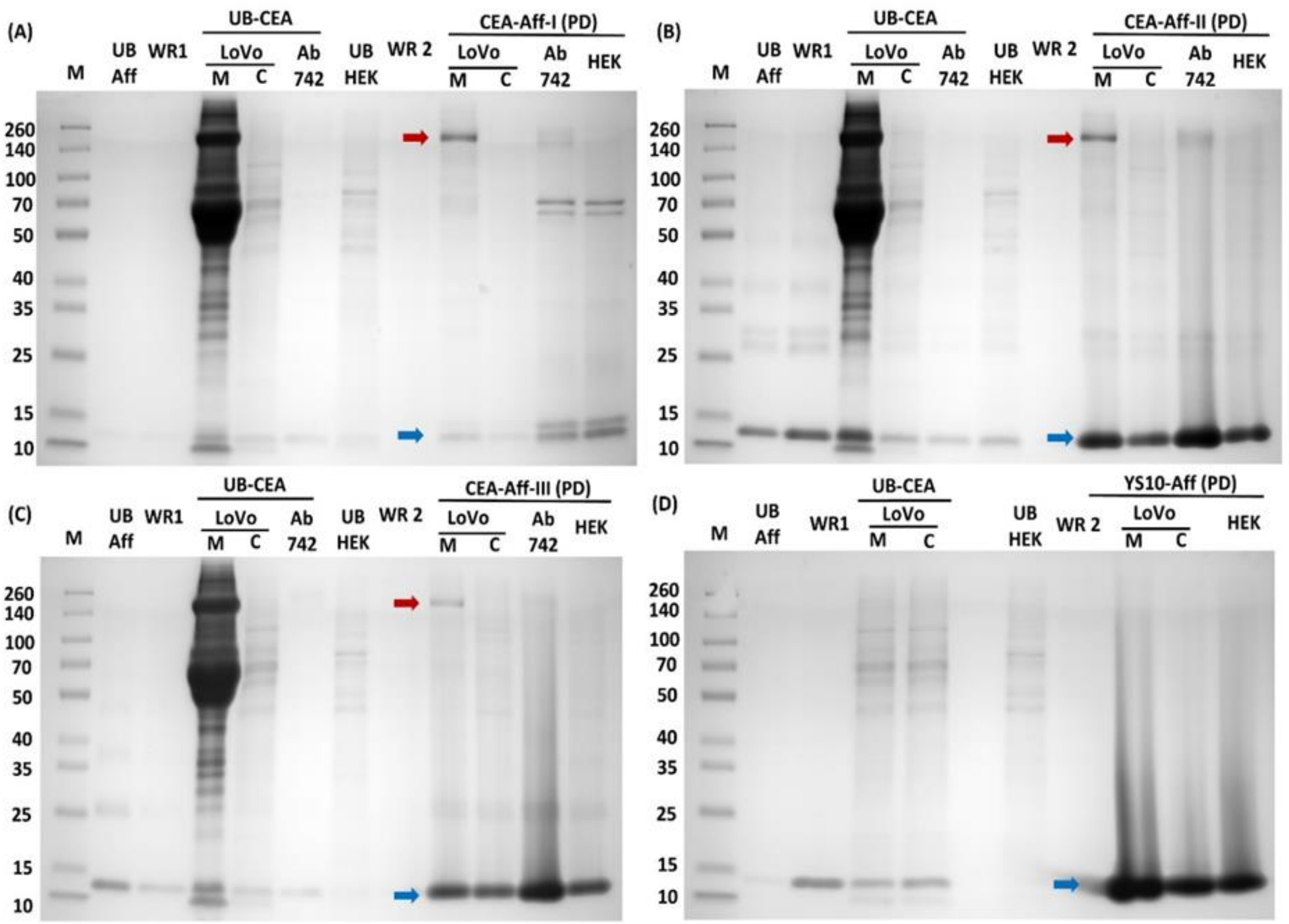

Supplementary Figure S1: Affinity-precipitation assay using anti-CEA Affimer as ligand to capture the CEA protein

Gels showing the fractions collected during the pull-down assay using CEA binding Affimer-I to III (A-C) as ligand to capture the CEA protein from the cell lysates of Lovo (LoVo C) and from the collected media (LoVo M). Yeast SUMO-10 binder was used as control Affimer (D), cell lysates of HEK 293 were used as negative control analyte and pure native human CEA from Abcam (Ab742) was used as positive control of CEA. WR 1 and 2 denote as collected fractions of wash solution and M denotes as protein marker.

Selection and characterisation of Affimers specific for CEA recognition

Shazana Hilda Shamsuddin[1,2*], David G. Jayne[5], Darren C. Tomlinson[3, 4], Michael J. McPherson[3, 4] and Paul A. Millner[1*]

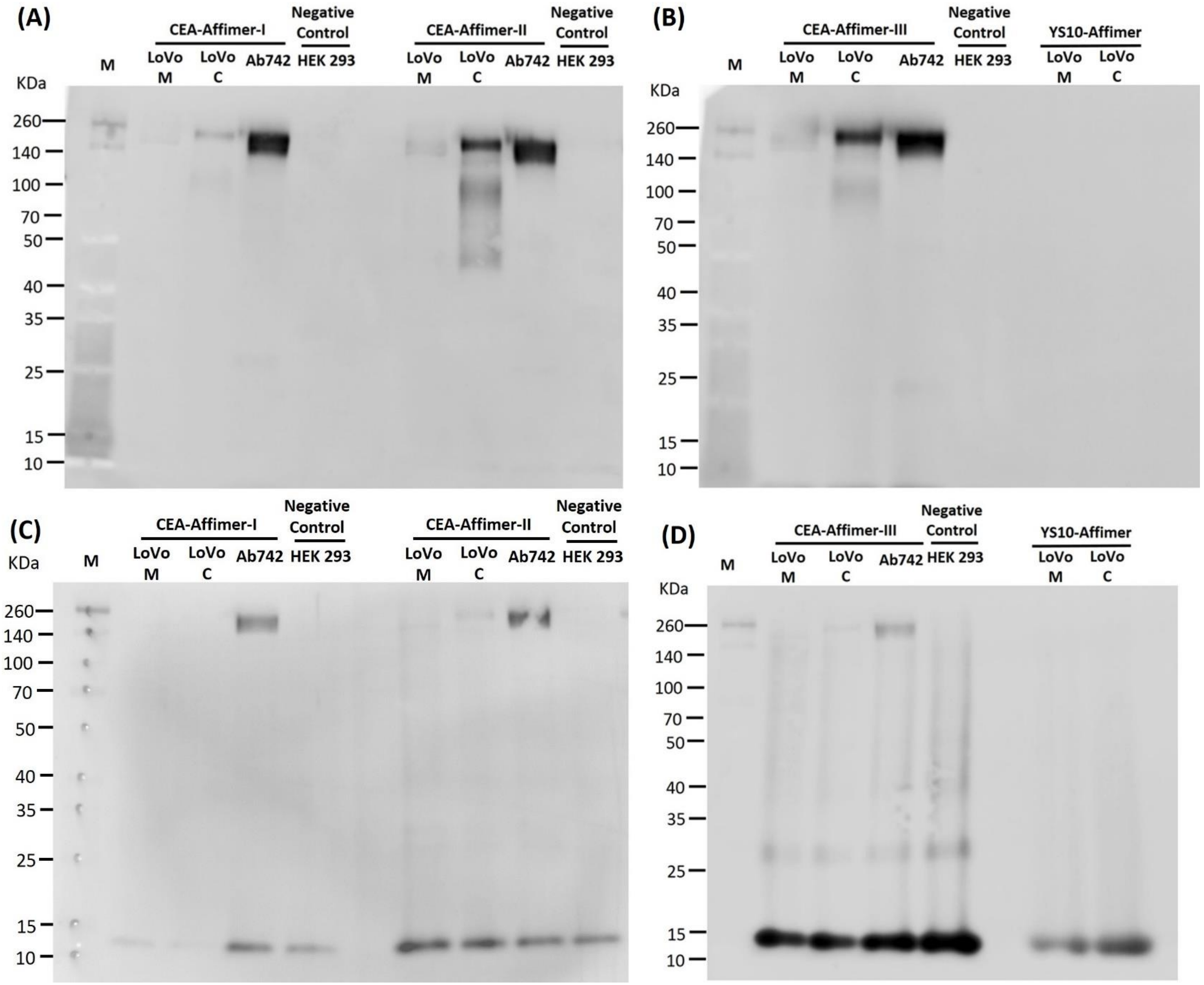

Supplementary Figure S2: Immunoblotting of Affimer precipitated CEA

Full-length blots showing the monoclonal anti-CEA and anti-His_6 tag antibodies were used to probe (A, B) the CEA protein and (C, D) Affimers, respectively from the pull-down complex. Yeast SUMO-10 binder was used as a control Affimer. Commercial CEA from Abcam was used as positive control and cell lysates from HEK 293 as negative control. LoVo M and C are samples from medium and cell lysates, respectively.

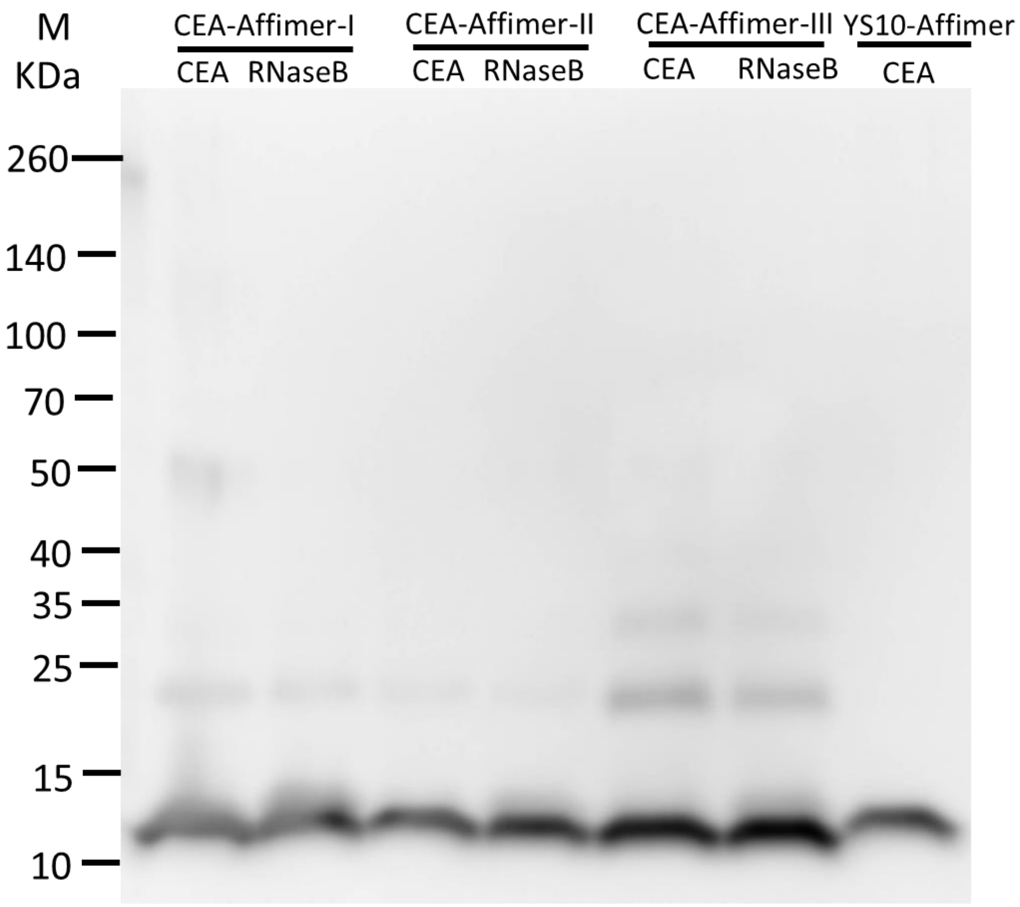

Selection and characterisation of Affimers specific for CEA recognition

Shazana Hilda Shamsuddin[1,2*], David G. Jayne[5], Darren C. Tomlinson[3, 4], Michael J. McPherson[3, 4] and Paul A. Millner[1*]

Supplementary Figure S3 : Immunoblotting of Affimer precipitated deglycosylated CEA

Immunoblotting of affinity-precipitation using CEA binding Affimers as ligand to pull-down deglycosylated CEA (n=2). Full-length blot showing the monoclonal anti-His_6 tag antibody probed the Affimers. Yeast SUMO-10 binder was used as a control Affimer and RNase B was used as non-specific analyte.

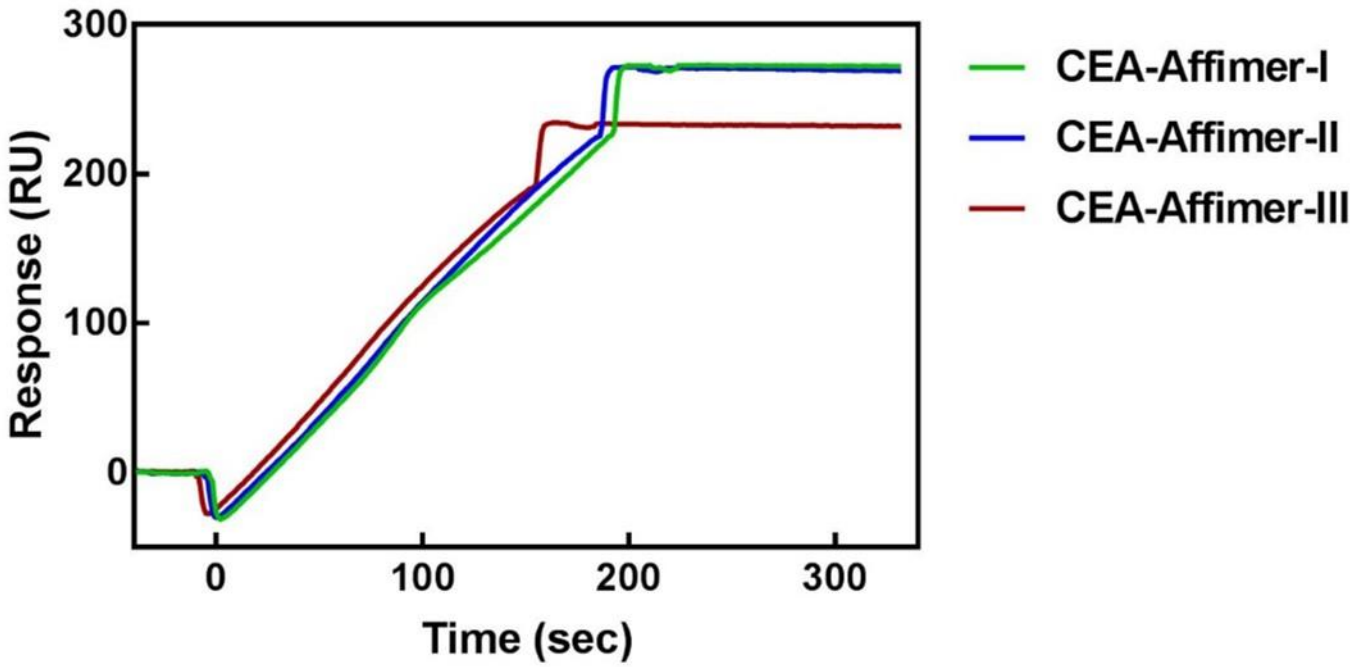

Selection and characterisation of Affimers specific for CEA recognition

Shazana Hilda Shamsuddin[1,2*], David G. Jayne[5], Darren C. Tomlinson[3, 4], Michael J. McPherson[3, 4] and Paul A. Millner[1*]

Supplementary Figure S4: Immobilization of biotinylated CEA binding Affimers on streptavidin coated SPR chip

Sensograms show immobilization of biotinylated anti-CEA Affimers onto streptavidin coated surface at low-density (~230-270 RU). The corrected response signals were obtained by subtracting the response from an unmodified reference surface.

Selection and characterisation of Affimers specific for CEA recognition

Shazana Hilda Shamsuddin[1,2*], David G. Jayne[5], Darren C. Tomlinson[3, 4], Michael J. McPherson[3, 4] and Paul A. Millner[1*]

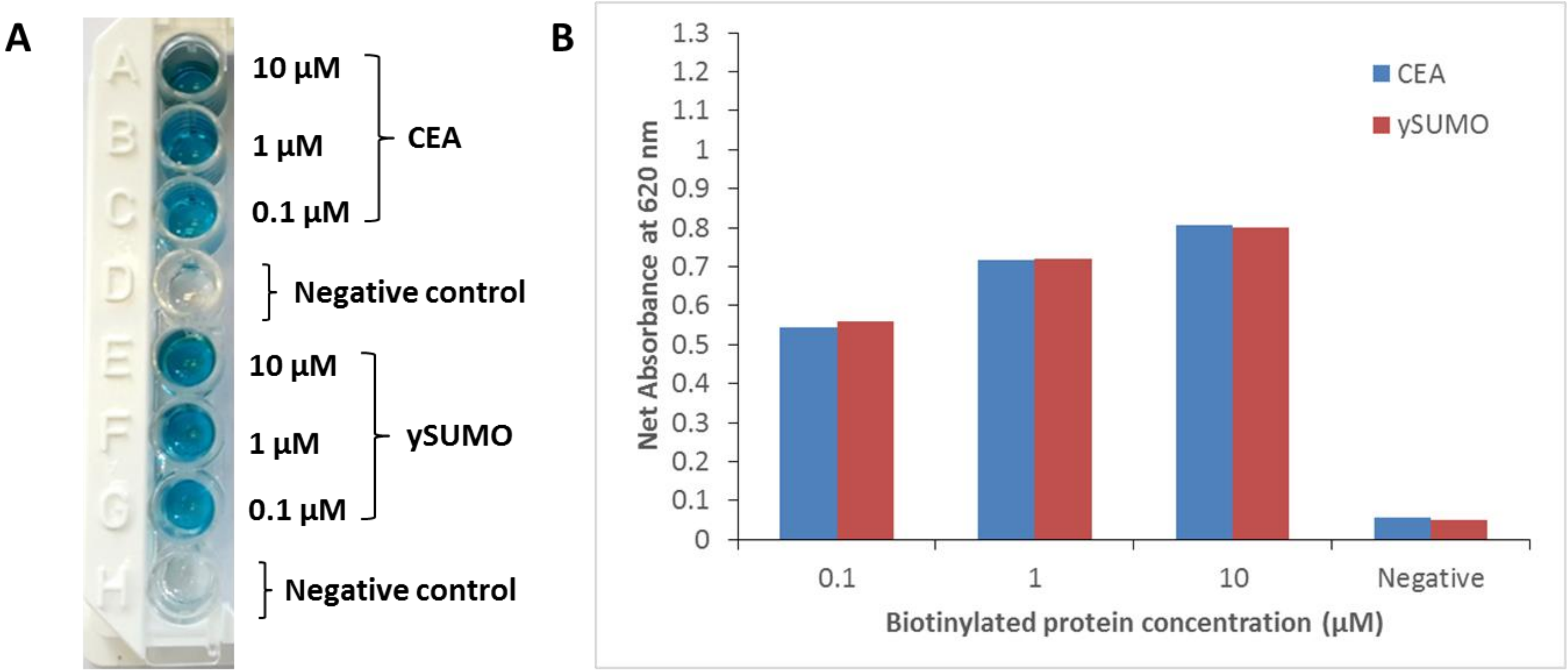

Supplementary Figure S5: ELISA of biotinylated CEA and ySUMO proteins

(A) Well A to C and E to G contained biotinylated CEA and ySUMO proteins, respectively, in decreasing concentration from 10 to 0.1 µM. Well D and H represent negative controls for each sample in which the biotinylated protein was omitted (B) Comparison between biotinylated CEA (blue bar) and ySUMO (red bar) at each analyte concentration are shown. ySUMO was used as positive control protein during panning process. Data is A_{620} using TMB as substrate.
